# Psychological Issues in Inflammatory Bowel Disease: An Overview

**DOI:** 10.1155/2012/106502

**Published:** 2012-06-21

**Authors:** M. S. Sajadinejad, K. Asgari, H. Molavi, M. Kalantari, P. Adibi

**Affiliations:** ^1^Department Psychology, College of Educational Science and Psychology, University of Isfahan, Isfahan, Iran; ^2^Integrative Functional Gastroenterology Research Center, Isfahan University of Medical Sciences, Isfahan, Iran

## Abstract

Inflammatory bowel disease (IBD) including Crohn's disease (CD) and ulcerative colitis (UC) is a chronic and disabling disease with unknown etiology. There have been some controversies regarding the role of psychological factors in the course of IBD. The purpose of this paper is to review that role. First the evidence on role of stress is reviewed focusing on perceived stress and patients' beliefs about it in triggering or exacerbating the course of IBD. The possible mechanisms by which stress could be translated into IBD symptoms, including changes in motor, sensory and secretory gastrointestinal function, increase intestinal permeability, and changes in the immune system are, then reviewed. The role of patients' concerns about psychological distress and their adjustment to disease, poor coping strategies, and some personality traits that are commonly associated with these diseases are introduced. The prevalence rate, the timing of onset, and the impact of anxiety and depression on health-related quality of life are then reviewed. Finally issues about illness behavior and the necessity of integrating psychological interventions with conventional treatment protocols are explained.

## 1. Introduction

Inflammatory Bowel Disease (IBD) describes a group of chronic gastrointestinal tract diseases that are relapsing and remitting; the term primarily comprises Crohn's disease (CD) and Ulcerative Colitis (UC). The prevalence of these diseases has increased in the past decades, up to 120–200/100000 and 50–200/100000 persons for UC and CD, respectively [1]. To date, there is no certain cure for IBD, and treatment is aimed at managing the inflammatory response during flares and maintaining remission with a focus on adhering to therapy [2]. The etiology of IBD is unknown, but genetic, immune, and environmental factors are each thought to play a role in its causation [1, 3, 4]. These factors interact together, so in a person who is predisposed genetically, environmental factors trigger immune dysfunction and bowel symptoms [5]. One of these environmental triggers may be psychological factors particularly psychological stress. 

## 2. Role of Psychological Stress in IBD 

A belief in the relevance of psychological factors to IBD is not new. Historically, it was first in the 1930s that gastroenterologists and psychiatrists suggested that emotional life events and experiences are likely related to exacerbation of intestinal symptoms [6]. At that time, IBD was considered as a psychosomatic disease, and its relation to stress and other psychological factors was thought so strong that researchers felt no need to use any control group in their studies. A few decades later, this finding was questioned mostly due to methodological weaknesses and uncontrolled studies published in this area. For a while IBD was considered as an organic disease, and psychological influences were discounted as contributing to it. But further anecdotal evidence and clinical observations indicated that stressful experiences could adversely affect the course of IBD. 

 Indeed many review articles have now emphasized the relationship between stress and IBD [6–10], concluding that confusions and controversies in published reports were partly due to differences in definitions of stress (e.g., stressful life events or hassles, daily stress) and partly due to inclusion of mixed groups of patients (CD versus UC) and/or mixed status of disease (active versus inactive) [6, 8]. Therefore, the major trends in recent studies were to differentiate between CD and UC patients, and to utilize the notion of perceived stress, which emphasizes on individual's subjective perception of stress and his/her emotional response to it [11].

 These trends have contributed to resolving controversies, and illuminating the role of psychological stress in IBD. Thus, while the role of stress in the onset of IBD has not been established, there is no doubt that stress is a triggering and exacerbating factor in relation to the course and symptoms of IBD [8, 10, 12, 13]. Indeed it can be considered as one of the determinants of disease relapse [12, 14, 15]. However, there are some discordant reports about a relation between stress and disease onset, like that of Li et al. [16] who, based on a follow-up study on the onset of IBD in parents who lost a child in Denmark, found a negative relationship between psychological stress and development of IBD. These conclusions provide support for the beliefs of almost 75% of patients with IBD that stress, or their own personality is a major contributor to the development of their disease [10, 12], and of more than 90% that it influences their disease activity [13, 17].

## 3. Possible Mechanisms of the Effects of ****Psychological Stress on Patients with IBD 

In the light of recent advances in psychobiological research, what are the mechanisms by which the course of IBD can be influenced by stress? 

### 3.1. Nonspecific Effects

 Many of the IBD symptoms experienced by patients may be due to stress-induced changes in gastrointestinal (GI) function. There is a richly innervated nerve plexus between the enteric nervous system (ENS) and its spinal and autonomic connections to the central nervous system, known as the brain-gut axis. GI motor, sensory and secretory function as well as thresholds for the perception of pain [13], can be affected by psychological and emotional stress directly or indirectly through this axis. These effects are mediated by substance P (SP), vasoactive intestinal protein (VIP) [18], several neuropeptides, neurotransmitters, and hormones [12, 19]. Stress stimulates the secretion of corticotropin-releasing factor (CRF) either from central or peripheral parts of CNS (hypothalamus and adrenal cortex, resp.). While central CRF regulates the ACTH-cortisol system, peripheral CRF directly influences gastrointestinal motility. Endogenous CRF mediates the stress-induced inhibition of the upper GI tract motility and stimulation of colonic motility [12, 20]. Thus when symptoms such as abdominal pain and change in bowel function occur in IBD without significant disease activity, they may be attributed, at least in some instances, to alterations in motor and sensory function as a result of psychological stress.

### 3.2. Intestinal Permeability

Psychological stress can also increase intestinal permeability, probably as a result of alterations in the cholinergic nervous system and mucosal mast cell function [21]. Söderholm and Perdue [22] pointed out that various types of physical and psychological stress have an impact on several components of intestinal barrier function such as increasing intestinal permeability and stimulating secretion of ions, water, mucus, and even IgA. This increased permeability in turn reduces mucosal barrier function and alters bacteria-host interaction [12, 23]. However, based mainly on animal studies, these observations are likely to play a role in the pathophysiology of human IBD. 

### 3.3. Immunological Mechanisms

Finally, stress is also likely to mediate its effect on IBD through the immune system [15, 19]. On the one hand, it is believed that an inadequately controlled response within the intestinal mucosa leads to inflammation in patients who are genetically predisposed to IBD. Dysfunction of the intestinal immune system and cross-reactivity of its cells against host epithelial cells have been implicated as major mechanisms by which the inflammatory response occurs [5]. On the other hand, it is increasingly recognized that the (hypothalamus-pituarity-adrenal) HPA axis, autonomic nervous system (ANS), and ENS can interact directly with the immune system. Cytokines are essential immune molecules in the pathogenesis of IBD [24, 25]. Many researchers [15, 20, 26, 27] reported that chronic or acute stress can alter profiles of cytokines (e.g., IL-1_*β*_, IL6, IL10, IL4, and TNF_*α*_) or of hormones such as cortisol, which may contribute to the pathophysiology of IBD. There is a bidirectional communication between neurons and mast cells within the gastrointestinal tract [28], and mucosal mast cells can be activated by stress [15, 29]. Stress-induced activation of mast cells, through release of mediators such as eicosanoids, serotonin, and IL6 could contribute to the pathogenesis of IBD [29].

### 3.4. Indirect Effects

**Figure 1:**
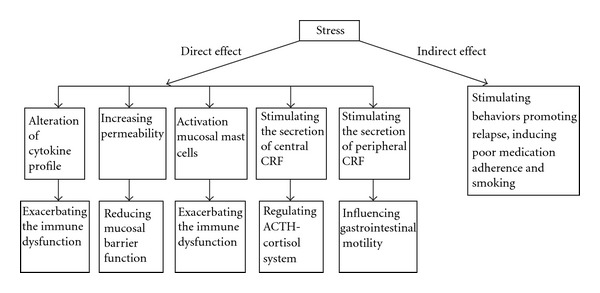
Direct and indirect mechanisms by which the course of IBD can be influenced by stress.

In addition to the above-mentioned direct pathways, stress can also indirectly affect clinical course of IBD. These indirect effects are exerted through behaviors known to promote relapse [14] and include poor medication adherence [30] and smoking [31]. Direct and indirect mechanisms by which the course of IBD can be influenced by stress are shown in Figure 1.

## 4. Coping with IBD 

Once IBD develops, the unpredictability, uncertainty, and chronic course of the disease can cause a wide range of psychological and interpersonal concerns to patients. These include loss of control of the bowel, fatigue, impairment of body image, a fear of sexual inadequacy, social isolation of dependency, a concern about not reaching to one's full potential, and feeling dirty [13, 32]. Indeed, symptoms, such as faecal incontinence or soiling and lack of bowel control, can lead to a loss of self-unworthiness or cause stigmatization in patients [33, 34]. 

Dealing with these concerns needs appropriate coping strategies and good adaptation. Unfortunately, however, a variety of studies suggest that IBD patients rely significantly on passive coping strategies [34, 35], utilizing fewer purposeful problem solving and positive reappraisal, and more escape-avoidance strategies [36, 37]. Concerns such as those listed above on the one hand, and passive and/or avoidant coping on the other hand, lead to psychological distress [38] with maladaptation and poor adjustment to the disease.

 Sewitch et al. [39] and Mikocka-Walus et al. [40] using Symptom Checklist-90-R (SCL-90) indicated that IBD patients had impaired psychological functioning. When patients receive a new diagnosis of IBD, a series of psychological adaptive steps occurs. For example, the patient may do an initial evaluation of the disease's likely impact on his/her life, subsequently showing emotional reactions such as distress, grief, and sometimes guilt, exhibiting a behavioral response involving taking new medications, seeking social support, and modifying their diet; various degrees of denial and or disease acceptance may occur. This adaptive process is complex; it is likely to be influenced by a range of factors including severity of disease, age of onset of disease, its extent of interference in the patient's life and future plans [32], beliefs and thoughts about illness and health, illness attribution [37], emotional status [41], and previous experiences.

 Among these, factors such as social support and affective state (in the frame of personality trait) have been studied in detail. Sewitch et al. [39] revealed that the relationship between psychological distress and perceived stress changes according to the level of satisfaction with social support. For patients who experienced moderate-to-high levels of perceived stress, high satisfaction with social support decreased the level of psychological distress and facilitated adjustment to the disease, a point which highlights the importance of social support in maintaining mental health in and adjustment to IBD. Moreover, Pellissier et al. [41] suggested that negative effect was associated with poorer coping to IBD.

## 5. Personality Traits

 Perhaps these factors can be integrated together and attributed to personality traits. Indeed, some patients with IBD believe that their own personality is a major contributor to the development of their disease [17]. In this context, Thornton and Andersen [42] suggest that personality traits can modulate the relationship between stress and the immunological reaction to it.

### 5.1. Neuroticism and Perfectionism

In IBD patients, the commonest personality trait is reported to be neuroticism [17, 43, 44]; furthermore, high neuroticism scores appear to reduce psychological wellbeing, psychological adjustment, and quality of life in patients with IBD [44, 45]. Another personality characteristic, emphasized in IBD patients, is perfectionism [46]; its negative impact in IBD is probably explained by its relationship with negative cognitive biases, heightened reactivity to stressors, and feeling pressured to be and look perfect. The latter may be particularly detrimental for IBD patients because these conditions are often accompanied by stigma, shame, feeling of dirty, and a burden [47]. The above investigators have shown a relationship between perfectionism and the psychological impact of IBD, so as the trait was associated with emotional preoccupation coping a maladaptive coping way with disease.

### 5.2. Alexithymia

Some studies have shown alexithymia to be another common personality characteristic in IBD patients. Patients with alexithymia have difficulty in recognizing and verbalizing emotions, and their ability to regulate emotions and express them to others is usually reduced [48]. Numerous studies [36, 44, 45, 49] have shown that IBD patients have higher scores for alexithymia than controls. In Jones, Wessinger, and Crowell's study [36], the scores of 74 IBS patients, 55 healthy control subjects, and 48 IBD patients compared on Toronto Alexithymia Scale and results showed that the IBS and IBD patients had higher scores on measures of alexithymia than controls but did not differ from one another. Porcelli et al. [49] in an epidemiological study compared 121 functional gastrointestinal disorders patients, 116 IBD patients, and a group of 112 healthy subjects using Toronto Alexithymia Scale. Their results showed that the FGID group was significantly more alexithymic than the IBD group, and the scores of two gastrointestinal groups were higher than the normal healthy group. Even after controlling for the influence of education, gender, anxiety, depression and gastrointestinal symptoms, these differences remained significant. Moreno-Jiménez et al. [44] did not use any control group. In their sample comprised of 60 UC and 60 CD patients, they have tried to address this question that, which personality factors may predict HRQOL in IBD patients. They showed that difficulty in describing one's feelings was significant on predicting two dimensions of HRQOL, that is, systemic symptoms and social functioning. Difficulty in describing one's feelings negatively predicted systemic symptoms and social functioning. Patients experiencing more difficulty in describing their feelings reported lower HRQOL. 

 However, Drossman and Ringel [13] reemphasized that while alexithymia is not specific to IBD, it may lead patients to communicate their psychological distress through somatic and behavioral symptoms rather than verbal communication; this might occur particularly when patients have limited perceived social support or personality traits such as introversion. Whether alexithymia is specific to IBD or not, it has been reported that affected patients have greater difficulty in describing their feelings to others, have poorer disease outcome, lower psychological functioning, and worse health-related quality of life [44, 46, 50].

Although discrete personality traits have been studied among IBD patients, no certain personality type matches this disease to date. It is recommended that future research considers discrete personality traits observed in these patients and integrates them in such a way that the traits will be addressed to new personality types such as type D [51, 52] and C [53], which are well matched with unregulated immune and hormonal systems that are characteristics of IBD.

## 6. Anxiety and Depression

The numerous concerns and worries mentioned above, together with patients' awareness of its incurability and uncertain course and prognosis, and their fear of surgery or the development of cancer, are all likely to contribute to a risk of anxiety in people with IBD [54, 55]. Once a patient develops IBD, he/she usually might form adaptive adjustment to it and accept the condition. Sometimes when patient has weak coping skills or social support or he/she may be personally predisposed (some personality trait such as neuroticism), he/she may feel frustrated, sad, and avoid social events. According to Seligman's theory [56], unpredictable and incurable course of disease impaired individual's belief about self-control [33] and self-efficacy [23, 32, 57] and thereby caused helplessness and predisposed the patient to depression. 

There are some controversies about the comorbidity of clinical anxiety and depression in IBD patients. While some researchers [38, 58, 59] found no evidence of any association between these psychiatric disorders and either UC or CD, others [60–63] confirmed that depression and anxiety are common in IBD patients. The prevalence of anxiety and/or depression has been estimated to be as high as 29–35% during remission [64] and 80% for anxiety and 60% for depression during relapse [65]. Robertson et al. [17] and Mikocka-Walus et al. [40] distinguished between these disorders and reported that anxiety is more prevalent than depression in IBD.

 Another source of controversy lies in the question of whether psychological disorders precede and/or follow after onset of the IBD? Some researchers have considered the psychological disorders as a consequence of a new diagnosis [6, 20] and emphasized that IBD is not caused by a psychological condition. However, Kurina et al. [66], using a database of linked hospital records abstracts, in a retrospective nested case-control study on 12499 patients (7268 UC and 5231 CD) and 800000 controls with minor medical conditions not related to the conditions of interest, found that both depression and anxiety preceded UC (but not CD) significantly more often than would be predicted by chance; the relationships were strongest when the mental conditions were diagnosed shortly before UC. However, these disorders were significantly more common after the diagnosis of CD, and UC was followed by anxiety, not by depression. In contrast, Tarter et al. [67] reported that anxiety prior to diagnosis was common, in CD, but found no significant antecedent psychological disorder in UC. These researchers studied 53 consecutive IBD patients including 26 CD and 27 UC patients and 28 healthy controls. In this study compared to normal controls, CD patients manifest an increased prevalence of anxiety, depression, and panic disorder occurring at any time in their life. Only panic disorder had an excess prevalence in CD relative to community dwelling normals prior to the time of disease onset. Individuals with UC did not demonstrate an increased prevalence of psychiatric disorder before or after disease onset. Mikocka-Walus et al. [7] suggested that it is difficult to reconcile these two divergent findings, as neither study was appropriately controlled. However, the sample size of the Kurina et al.'s [66] group was substantially larger than of the Tarter et al. [67], and it is a methodological strength that partly facilitates the conclusion. 

Whether anxiety and depression appear before or after the onset of IBD, physiological data [68, 69], suggest that these mood disorders can stimulate production of proinflammatory cytokines and thereby adversely affect the course of IBD, a conclusion supported by clinical observations [64]. Drossman and Ringel. [13] suggest that psychological disturbances as a component of the illness may modulate its clinical expression, rather than being etiologic or specific to IBD. It is, therefore, a priority to pay careful attention to the possibility of mood disorders in patients with IBD.

## 7. Quality of Life (QOL)

IBD generally begins in child- or young adulthood and lasts life-long. Although the life expectancy of IBD patients approximates to that of healthy people, IBD substantially impairs patients' health-related quality of life (HRQOL) [70, 71]. Relevant factors include those discussed above, the chronicity of IBD, its complications, associated physician visits and hospitalizations, and side effects of medical treatment or surgery. It is not surprising, therefore, those patients with active IBD have poorer disease-specific quality of life relative than those with inactive disease [35, 72–74]. Of course, poor HRQOL is not restricted to active episodes, and the negative impact of IBD on patients quality of life continues even when it is inactive. Considering disease type, Mikocka-Walus et al. [40] and Farmer et al. [75] pointed out that impairment in psychosocial dimensions of HRQOL is greater for CD than UC. Mikocka-Walus et al. [40] compared psychological problems such as anxiety and depression and impairment of quality of life in IBD, IBS and Hepatitis C, and general population. These researchers found that each of three groups had significantly lower quality of life than the general population. In IBD group, 31 participants with CD had poorer physical quality of life in Physical Component Summary (PSC) subscales of SF-12 than 33 UC patients (*P* = 0.016). Farmer et al. [75], using an interview consisted of four categories: functional/economic, social/recreational, affect/life in general, and medical/symptoms, studied quality of life on 94 patients with ulcerative colitis and 70 patients with Crohn's disease. They found that Patients with ulcerative colitis had better scores on medical/symptoms' category in the interview than those with Crohn's disease. These researchers believed that perhaps these findings could be attributable to experiencing more severe disease by CD patients than UC patients. Drossman and Ringel [13] have pointed out to this conclusion, as well. However, others have suggested that after severity of disease was taken into account, there were no significant differences between CD and UC in terms of HRQOL [57, 70]. 

However, the physical symptoms of IBD do not completely explain the reductions of HRQOL in affected patients because disease activity and the intensity of patients' symptoms do not significantly correlate with their subjective impairments [77]. Indeed, sociodemographic variables influence quality of life. For example, Sainsbury and Heatley [35], Casellas et al. [70], and Haapamaki [71] listed the effects of gender (women had poorer QOL than men), level of education, socioeconomic status, and older age on HRQOL in IBD patients. In addition to sociodemographic variables, others have noted that psychological factors can also affect HRQOL in these diseases. In this regard, more psychological disturbance and the presence of anxiety or depression contribute to poorer HRQOL, regardless of severity of the IBD [43, 59, 74]. Furthermore, Moreno-Jiménez et al. [44] and Boye et al. [45] suggest that factors such as personality traits may influence psychological well-being and HRQOL; in their studies, neuroticism and greater difficulty in describing feelings to others were related to poorer HRQOL. Overall, because of its influence on patients' psychological well-being, social adjustment to their IBD and health care utilization, integrating HRQOL into the routine clinical assessment of patients with IBD, and targeting it as a treatment aim is strongly recommended. The factors affecting QOL of patients with IBD summarized in Table 1.

## 8. Illness Behavior

The personal meaning of the illness, the individual's attitudes and expectations, and illness attributions to internal or external factors may all affect disease-related concerns and consequently a patient's adjustment to an illness. Since patients differ in social context, cultural heritage, value systems, family structure, prior experiences with illness, and psychological status, each individual is likely to respond differently to the challenge of a chronic illness such as IBD. Levenstein et al. [78] suggested that because of the impact of sociocultural beliefs and values about the illness, the impact of a given disease may also vary significantly between one country and another, even if its biological behavior is uniform. 

 For example, a patient with ulcerative colitis who suffers from abdominal pain may not go to the physician if he/she has previously experienced those symptoms without serious consequences, or if he/she has grown up in a family where attention to illness has been minimal, or if he/she believes that complaining might be regarded as weakness. Another patient with the same disease activity and symptoms may frequently utilize healthcare services if he/she perceives symptoms to have dangerous consequences and is seeking disability or comes from a family where greater attention has been paid to illness. In this context, Drossman et al. [79] found that the number of physician visits in IBD patients was related to both psychological and physical health factors, so that the presence of psychological distress such as depression may lead to more frequent physician visits.

For the above reasons, physicians need to establish a close and effective therapeutic relationship with their patients, be sensitive and responsive to their patients' concerns about IBD, provide information consistent with the patients' questions and needs, and educate their patients properly about their condition and planned treatment, thereby reducing patients' concerns and uncertainties and their dependency on the healthcare system.

## 9. Management of Psychological Disorders****in Patients with IBD

As mentioned earlier, psychological disorders such as anxiety and depression are common among IBD patients. Even if the severity of these psychological problems does not reach the clinical definition of psychiatric disease, psychological distress, concerns, worries, fears, and poor coping strategies which may lead to reduced quality of life fully justify professional attention. Most current conventional medical treatments for IBD are associated with potential side effects, some of which are psychological (e.g., mood changes, mania, or depression induced by corticosteroids), and none of them pay attention to patient's psychological status or concerns or QOL [63]. Therefore, integrating psychological treatment with conventional medical therapy to improve psychological distress and coping strategies and when necessary to alleviate depression or anxiety is likely to be beneficial. 

### 9.1. Effect of Psychological Interventions on IBD Activity

It has been suggested that if psychological stress, for example, by worsening mucosal barrier and immune function, is a pathogenic factor in IBD (see above), then psychological intervention aimed at stress reduction and may potentially reduce disease activity [9, 15]. Specifically, Niess et al. [19] and Thornton and Andersen [42] proposed that psychological interventions such as relaxation training influence stress-mediated alterations of the immune system. Furthermore, psychological interventions can reportedly improve the course of some immune-mediated diseases such as cancer and HIV [93, 94]. More work is needed to assess the proposal that psychological approaches could affect the course of IBD itself. 

### 9.2. Improving Personal Control

Another reason for incorporating these interventions as complementary options in the treatment protocol relates to establishing and highlighting for the patient a sense of personal control. Compared to medical therapy that emphasizes patient's obedience to their doctor in relation, for example, to medication adherence, psychological interventions engage the patient in the treatment process and increase a sense of personal control of their illness. These interventions do this by educating patients about cues for managing stress and relaxing them, assist the patients to solve their problems rather than avoiding them or surrendering them to others, and restructuring their cognitions rather than trying to alter their external environment. Many researchers [95] in sociology and psychology have indicated that personal control is important to psychological functioning and can be regarded as a robust predictor of physical and mental well-being. Furthermore, psychological interventions may increase self-efficacy in patients and thereby improve their capacity for coping and managing their distress. 

### 9.3. Potential Psychological Therapies

Numerous psychological interventions have been developed and studied in IBD, with a range of outcomes. Keller et al. [80] and Wietersheim et al. [81] reported that supportive-expressive and psychodynamic therapy may be ineffective in the treatment of psychological comorbidities and somatic course of IBD. Some studies [43, 82–86] showed that although cognitive-behavioral therapy or stress management may lead to significant improvements in anxiety, depression, and quality of life, they have no effect on the course of patients' IBD. In contrast, others [87–89] suggested that stress management, relaxation training, and IBD-focused counseling have been useful both for psychological problems and the clinical symptoms of IBD. A comprehensive lifestyle modification program [90] and mind-body therapy [91] have also been studied in IBD patients and revealed significant improvements in quality of life, anxiety, and psychological well-being.

### 9.4. Antidepressants

High prevalence of psychological disorders, such as anxiety and depression in patients with IBD, may recommend psychopharmacological therapies specially antidepressants as an alternative treatment for these patients. Although a qualitative study [92], based on interviewing with gastroenterologists, showed that some gastroenterologists use antidepressants for treating pain, anxiety and/or depression, and insomnia in patients with IBD, to date application of these drugs is not straightforward. Mikocka-Walus et al. [96], based on their systematic review about antidepressants and IBD, reported that tricyclic antidepressants (e.g., Amitriptylin, dothiepin, prothiaden, doxepin, imipramin, and nortriptyline) not only alleviate psychological distress, but also have some positive effects on somatic status of IBD patients through reducing pain, gut irritability, and urgency of defecation. Newer antidepressants are not prescribed as much as TCAs by physicians. Recent systematic review by Mikocka-Walus et al. [97] even reported that antidepressants have positive effect on inflammation of the bowel in IBD patients. Since the most published data in this area have not been randomized and have methodological weaknesses, Hardt et al. [63] concluded that it is impossible to make a definitive statement of efficacy of antidepressants on mental and somatic status of IBD patients. 

Overall, while there are contradictory reports on the effect of psychological interventions on clinical course of IBD, most show a positive effect on psychological and emotional status and HRQOL. Contradictory findings may be in part due to difference in trial design, the components of the tested treatment protocols, outcome measures assessed, and other confounding variables. Undoubtedly, more rigorous better designed studies in this field are needed. With regard to recent findings about the effect of psychological interventions on immune modification [97, 98] and reducing the likelihood of relapse [57], the necessity of incorporating them to conventional treatment protocol of IBD is more illustrated. Options for management of psychological disorders in patients with IBD are listed in Table 2.

## 10. Conclusion

Current evidence indicates that psychological factors play a role both in the pathophysiology and course of IBD and in how patients deal with these chronic and disabling diseases. Over the two last decades, improvements in study design and methodology, along with advances in psychoneuroendocrinology and psychoneuroimmunology, have led to improved, if still incomplete, understanding of the relevant pathophysiological mechanisms. The contribution of some psychological factors such as predisposing personality remains uncertain and requires further study. Most importantly, in view of the importance of psychological dysfunction in modifying illness behavior and its negative impact on symptoms and QOL, the integration of psychological interventions into conventional medical therapy, seems advisable. Where further study is most urgently needed, however, it lies in the analysis of the precise effects of these interventions on not only psychological state and quality of life, but also on the physiological parameters and the course of IBD itself. Such research should investigate also which is the most effective component, or combination of components for the psychological management of IBD.

## Figures and Tables

**Table 1:** Factors affecting QOL in patients with IBD.

	Factor	Study	Result
Disease-related factors	Disease activity	Vidal et al. [74]	Disease activity was one of strongest predictors of QOL impairments.
		Albersnagel and Dijkstra [72]	Disease activity adversely affects QOL of the patients.
		Haapamaki [71]	Disease activity was the most factors related to QOL impairment.
		Graff et al. [73]	Patients with active disease had poorer QOL scores, but participants with either active or inactive disease had suboptimal general QOL.
	Disease severity	Guthrie et al. [58]	Disease severity was one of factors contributed to impaired QOL.
	Disease type	Mikocka-Walus et al. [40]	CD patients tended to have poorer physical QOL than UC patients.
		Casellas et al. [70]	Disease type did not predict QOL scores.
		Guthrie et al. [58]	After controlling disease severity, there were no significant differences between CD and UC in QOL scores.
		Graff et al. [73]	Disease type was not contributor to QOL.
	History of surgery	Haapamaki [71]	Lower QOL scores were seen in those patients with a history of surgery.
	Disease chronicity	Haapamaki [71]	Lower QOL scores were seen in newly diagnosed patients.
		Casellas et al. [70]	Longer disease duration and lower recurrence/year index predicted a better QOL.
	Need for hospitalization	Casellas et al. [70]	Nonnecessity of hospitalization predicted a better QOL.

Demographic factors	Gender	Haapamaki [71]	Female had poorer QOL than men.
		Casellas et al. [70]	Female gender predicted a better QOL.
	Age	Haapamaki [71]	Older age patients had poorer QOL.
	Educational level	Casellas et al. [70]	Higher level of education predicted a better QOL.

Psychological factors	Personality	Vidal et al. [74]	Personality traits did not play a significant role in QOL.
		Moreno-Jiménez et al. [44]	Neuroticism and greater difficulty in describing feelings to others were related to poorer QOL.
	Psychological distress	Vidal et al. [74]	Psychological distress was one of strongest predictors of QOL impairments.
		Guthrie et al. [58]	Psychological symptoms were one of factors contributed to impaired QOL.
	Social support	Moradkhani [76]	Activity in online support groups was not related to QOL.

**Table 2:** Options for management of psychological disorders in patients with IBD.

Treatment	Study	Effectivity		
		Psychological problems	Course of IBD	Quality of life
Supportive-expressive and psychodynamic therapy	Keller et al. [80] and Wietersheim et al. [81]	Ineffective	Ineffective	Not reported
CBT or stress management	Boye et al. [45, 82], Sibaja et al. [83], Schwarz and Blanchard [84], Mussell et al. [85], Szigethy et al. [86]	Effective	Ineffective	Effective
	Garcia-Vega and Fernandez-Rodriguez [87], and Shaw and Ehrlich [88]	Effective	Effective	Not reported
IBD-focused counseling	Wahed et al. [89]	Effective	Effective	Not reported
Lifestyle modification program	Langhorst et al. [26, 90]	Effective	Not reported	Effective
Mind-body therapy	Elsenbruch et al. [91]	Effective	Not reported	Effective
Antidepressants	Mikocka-Walus et al. [7, 92]	Effective	Controversial	Not reported
